# Pan-cancer analysis of PDGFRB: Laying the foundation for the development of targeted immunotherapy drugs

**DOI:** 10.1097/MD.0000000000041797

**Published:** 2025-03-21

**Authors:** Qian Gao, Yan Cui, Feng Gao, Yan Yang, Weizhong Huangfu, Minjie Wang

**Affiliations:** a Medical Experiment Center, School of Basic Medicine, Inner Mongolia Medical University, Key Laboratory of Quality Research and Efficacy Evaluation of Traditional Chinese Medicine (Mongolian Medicine), Inner Mongolia Medical University, Huhhot, China; b School of Humanities Education, Inner Mongolia Medical University, Huhhot, China; c School of Pharmacy, Inner Mongolia Medical University, Huhhot, China; d The Affiliated Hospital of Inner Mongolia Medical University (Inner Mongolia Institute of Cardiovascular Diseases), Huhhot, China.

**Keywords:** cancer, immune checkpoint, PDGFRB, TCGA

## Abstract

PDGFRB is a type III tyrosine-protein kinase that is abnormally expressed in various cancers and can serve as a biomarker for cancer prognosis, as studies have demonstrated. However, a pan-cancer analysis of PDGFRB has not yet been carried out. The Cancer Genome Atlas (TCGA) and Genotype-Tissue Expression (GTEx) databases were utilized to analyze PDGFRB expression levels. Differential expression of PDGFRB in standard, tumor, and different clinical stage samples was calculated using R software (version 3.6.4). Immunohistochemical staining for Cholangiocarcinoma (CHOL) and Esophageal carcinoma (ESCA) was conducted on clinical patient samples. High-quality prognostic datasets from TCGA have been published in previous studies. Additionally, the TARGET follow-up data were obtained as supplementary information, excluding models with a follow-up period of less than 30 days. After conducting a rain analysis of PDGFRB, Kaplan–Meier and univariate Cox regression analyses were performed using the R software package. The DNA tumor stemness scores, derived from methylation signatures for each tumor, were obtained from previous studies. Finally, the infiltration of immune cells was analyzed, and the Pearson correlation between PDGFRB and five immune pathway marker genes was assessed. PDGFRB exhibited differential expression across most tumor types in TCGA, indicating a correlation with poor survival outcomes. The expression of PDGFRB influences the regulation of the immune system and is closely associated with immune cell infiltration, immune checkpoints, immune-activating genes, immune suppressor genes, chemokines, and chemokine receptors. PDGFRB is a cancer gene closely associated with prognosis and immunity in cancer patients, and it may serve as an immune checkpoint.

## 1. Introduction

Cancer occurs as one of the leading causes of mortality worldwide, and the annual count of cancer cases is growing. According to data from the National Cancer Center, in 2020, there were 19.29 million new instances of cancer globally, along with 9.96 million deaths from the disease.^[1]^ Traditional cancer treatment procedures include surgery, radiation, chemotherapy, and interventional therapies, etc. However, these treatment methods still have limitations, such as the feasibility of surgical treatment for solitary tumors and the possibility of inadequate tumor removal. Furthermore, the poor targeting of radiation and chemotherapy makes it difficult to differentiate cancer cells from normal cells, resulting in extensive side effects.

Immunotherapy is an emerging strategy for cancer treatment, and interleukin-2 (IL-2) is the first immunotherapy component used to treat malignancies.^[2]^ It can destroy tumors to prevent cancer and create a tumor microenvironment to assist in cancer formation.^[3]^ We must develop more effective strategies to control the immune system so that it can actively combat tumor growth and prevent the onset of cancer.

Platelet-derived growth factor receptor-β (PDGFRB) is a type III tyrosine protein kinase, mostly an isotype or heterodimer linked by disulfide bonds, which can only bind to PDGF-B.^[4,5]^ The body controls the migration, recruitment, and proliferation of smooth muscle cells, fibroblasts, and pericytes to ensure stable development.^[6]^ Numerous investigations have demonstrated that overexpression of PDGFRB raises the risk of cancer. For instance, high PDGFRB expression has been found in glioblastoma and invasive triple-negative breast cancer.^[7,8]^ In addition, studies have found that PDGFRB is mainly expressed by mesenchymal cells in epithelial tumors, and PDGFRB signals contribute to invasion and liver metastasis.^[9,10]^ In addition to the increased risk of cancer caused by PDGFRB overexpression, mutations in PDGFRB can also lead to cancer. For example, some spindle brain aneurysms are caused by mutations in PDGFRB.^[11]^ Some studies have compared the frequency of PDGFRB mutations in different cancers and found that the incidence of PDGFRB mutations was the highest in single-center Castleman disease.^[12]^ At present, PDGFRB is often fused with other genes for the study of some cancers, especially leukemia. Studies have shown that EBF1-PDGFRB can drive leukemia through TM-dependent loss of transcription factor function, increased proliferation, and synergistic interaction with other genetic impairments.^[13]^

PDGFRB has been shown to be a prognostic biomarker for gastric cancer,^[14]^ but there is no holistic pan-cancer study on PDGFRB. In this study, we investigated the pan-cancer expression of PDGFRB and its association with prognosis in cancer patients. Additionally, the relationship between PDGFRB and immune-related expression was investigated.

## 2. Methods

### 2.1. Data collection in PDGFRB pan-cancer analysis

The unified and standardized pan-cancer dataset was downloaded from UCSC (https://xenabrowser.net/): TCGA TARGET GTEx (PANCAN, N = 19,131, G = 60,499), and further extracted the gene data of PDGFRB (ENSG00000113721) in various tumors and normal tissues with the help of Sangerbox platform.^[15]^

### 2.2. Differential analysis of pan-cancer expression of PDGFRB

Samples from various cancers were screened for PDGFRB, and their expression levels were transformed accordingly. The difference in PDGFRB expression in standard samples, tumor samples, and samples at different clinical stages was calculated using R software (version 3.6.4).

For paired tissues, immunohistochemical staining was performed using paraffin wax sections of cancerous and para-cancerous tissues from patients with Cholangiocarcinoma (CHOL) and Esophageal carcinoma (ESCA). PDGFRB antibodies were obtained from BOSTER (Catalog Number: A00096-1).

### 2.3. Prognostic analysis of PDGFRB

A high-value prognostic dataset from the TCGA was obtained from a study published in the Cell Journal.^[16]^ The UCSC cancer browser was used to obtain the TARGET with a short visit data supplement and follow-up time in samples of 30 days. The Kaplan–Meier analysis was conducted using the R software package to evaluate the overall survival of patients in the TCGA cohort. Additionally, a univariate Cox regression analysis was performed with the R software package to determine the significance of PDGFRB in predicting overall survival (OS), disease-specific survival (DSS), disease-free interval (DFI), and progression-free interval (PFI) across various types of cancer.^[17]^

### 2.4. Tumor stemness analysis of PDGFRB

From this database, the expression data of PDGFRB in different samples was retrieved, and also tested samples from primary blood-derived cancer-peripheral blood, and primary tumors. The DNAss tumor stemness score was then calculated using the methylation characteristics of each tumor from the previous study.^[18]^

### 2.5. Immune infiltration analysis of PDGFRB

Each patient’s immune cell infiltration score in each tumor was reassessed by gene expression using R software. Pearson correlation analysis of PDGFRB and 5 immune pathway marker genes was performed using data derived from the TCGA database.^[19]^

### 2.6. Data analysis

Unpaired Wilcoxon Rank Sum and Signed Rank Tests were used for significance analysis, and analysis of variance (ANOVA) was used for different tests of multiple groups of samples. Unpaired Student *t* test was used to analyze the significance of PDGFRB differences between pairs, and R software was used for statistical analysis. *P < .05, **P < .01, ***P < .001, ****P < .0001; −, not significant.

### 2.7. Ethical approval

This article does not contain any studies with animals performed by any of the authors. The clinical samples have been approved by the participants. This study was approved by the “Inner Mongolia Medical University Medical Ethics Committee” on March 2, 2022, with approval number YKD202201143.

## 3. Results

### 3.1. Differential analysis of PDGFRB gene expression in pan-cancer

#### 3.1.1. Comprehensive analysis of PDGFRB expression across various cancers using TCGA and GTEx databases

**Figure 1.**
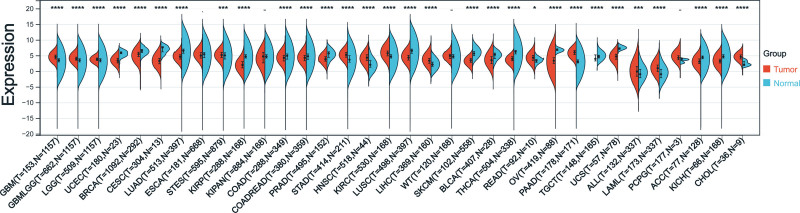
Analysis of differences in PDGFRB expression in various tumors using data from the TCGA and GTEx databases. GTEx = Genotype-Tissue Expression, PDGFRB = platelet-derived growth factor receptor-β, TCGA = The Cancer Genome Atlas.

Cancers with fewer than three samples of any type were excluded after screening and transforming data from the TCGA and GTEx databases. Subsequently, expression data was collected for 34 different types of cancer. Thirteen tumors showed significant up-regulation in PDGFRB expression, as calculated using R software to compare the expression levels between normal and tumor samples, (Glioblastoma multiforme (GBM), Glioma (GBMLGG), Brain lower grade glioma (LGG), Stomach and esophageal carcinoma (STES), Stomach adenocarcinoma (STAD), Head and neck squamous cell carcinoma (HNSC), Kidney renal clear cell carcinoma (KIRC), Liver hepatocellular carcinoma (LIHC), Rectum adenocarcinoma (READ), Pancreatic adenocarcinoma (PAAD), Acute lymphoblastic leukemia (ALL), Acute myeloid leukemia (LAML), and CHOL)). A notable decrease was observed in 17 tumors (Uterine corpus endometrial carcinoma (UCEC), Breast invasive carcinoma (BRCA), Cervical squamous cell carcinoma and endocervical adenocarcinoma (CESC), Lung adenocarcinoma (LUAD), Kidney renal papillary cell carcinoma (KIRP), Colon adenocarcinoma (COAD), Colon adenocarcinoma/Rectum adenocarcinoma esophageal carcinoma (COADREAD), Rectum adenocarcinoma (PRAD), Lung squamous cell carcinoma (LUSC), Skin cutaneous melanoma (SKCM), Bladder urothelial carcinoma (BLCA), Thyroid carcinoma (THCA), Ovarian serous cystadenocarcinoma (OV), Testicular germ cell tumors (TGCT), Uterine carcinosarcoma (UCS), Adrenocortical carcinoma (ACC), and Kidney chromophobe (KICH)) (Fig. 1).

#### 3.1.2. Analysis of PDGFRB expression in cancer samples across clinical stages

**Figure 2.**
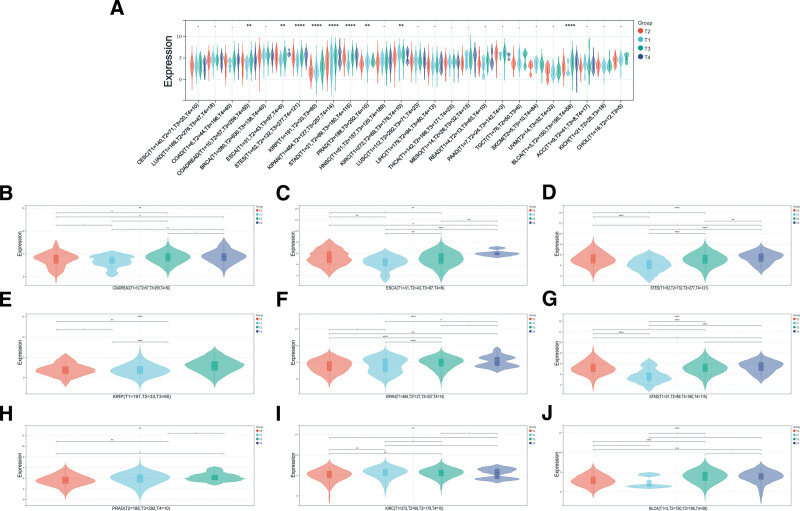
Pan-neoplastic expression of PDGFRB across various clinical stages (A). Nine cancer types show significant differences at different clinical stages (B–J). PDGFRB = platelet-derived growth factor receptor-β.

The data was screened and transformed utilizing the TCGA database. Cancer types with fewer than three samples were excluded, resulting in expression data for 26 distinct cancer types. R software was employed to analyze gene expression differences across various clinical stages of each tumor. This analysis identified significant differences in nine tumor types (COADREAD, ESCA, STES, KIRP, KIPAN (Pan-kidney cohort (KICH + KIRC + KIRP)), STAD, PRAD, KIRC, and BLCA) (Fig. 2).

#### 3.1.3. Differential expression of PDGFRB in cancer and adjacent normal tissues

**Figure 3.**
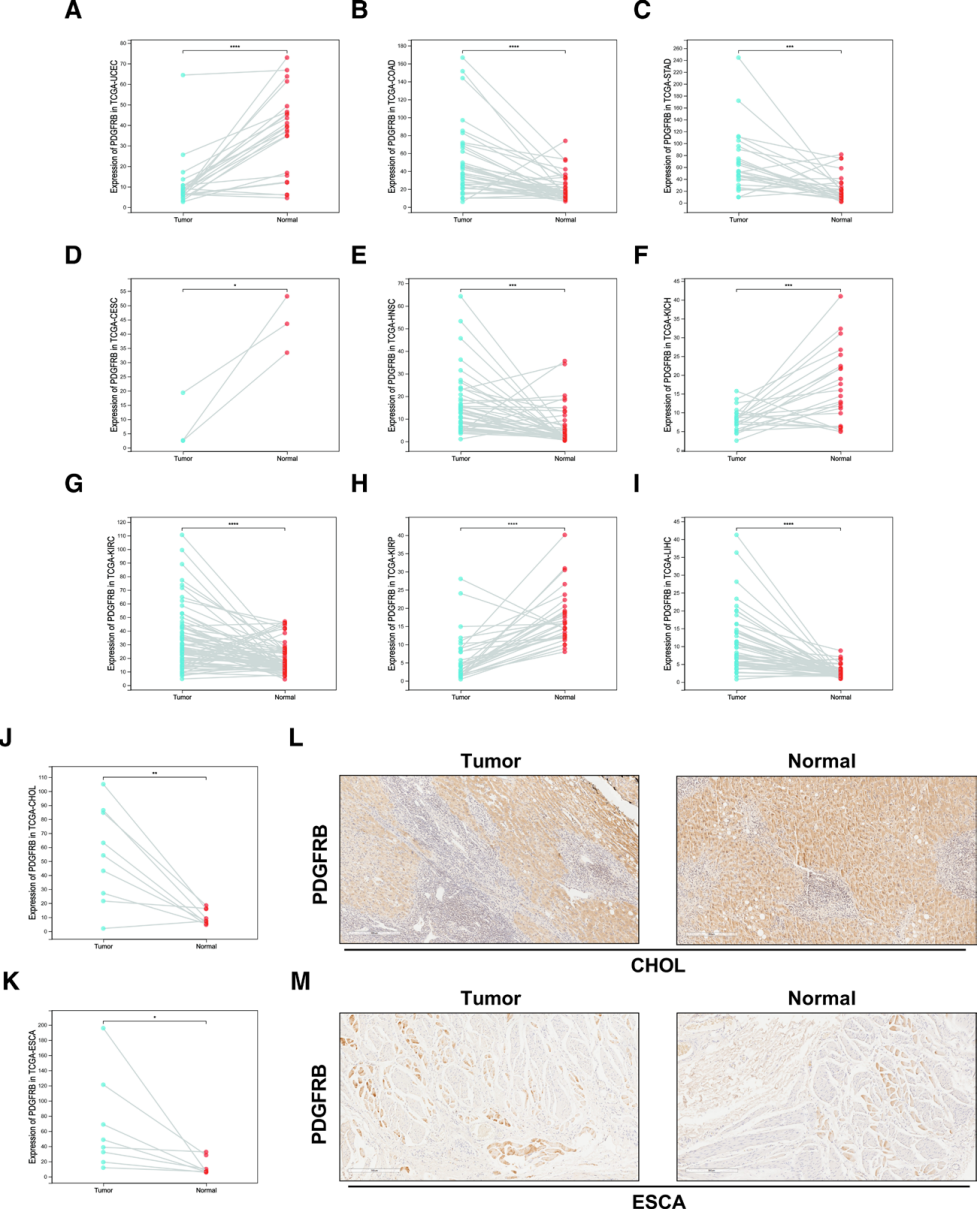
Pairwise analysis of PDGFRB in cancer (A–M). Paired expression in 11 Cancers with significant differences in PDGFRB (A–K). Immunohistochemical staining of paired human cancer tissues from CHOL and ESCA (L–M). CHOL = cholangiocarcinoma, ESCA = esophageal carcinoma, PDGFRB = platelet-derived growth factor receptor-β.

For paired tumors and normal tissues from the TCGA database, a total of 42 cancer samples for the PDGFRB gene were initially screened. After removing samples with incomplete data, 21 cancer samples remained. The results indicated significant differences in PDGFRB expression across 11 types of cancer (UCEC, ESCA, COAD, STAD, CHOL, CESC, HNSC, KICH, KIRC, KIRP, LICH)) (Fig. 3A–K). PDGFRB expression was clearly observed in the pathological sections of CHOL and ESCA, with significant differences observed between normal and cancerous tissues (Fig. 3L and M).

### 4. Prognostic significance of PDGFRB

#### 4.1. Differential analysis of PDGFRB in the survival rate of different cancer patients

**Figure 4.**
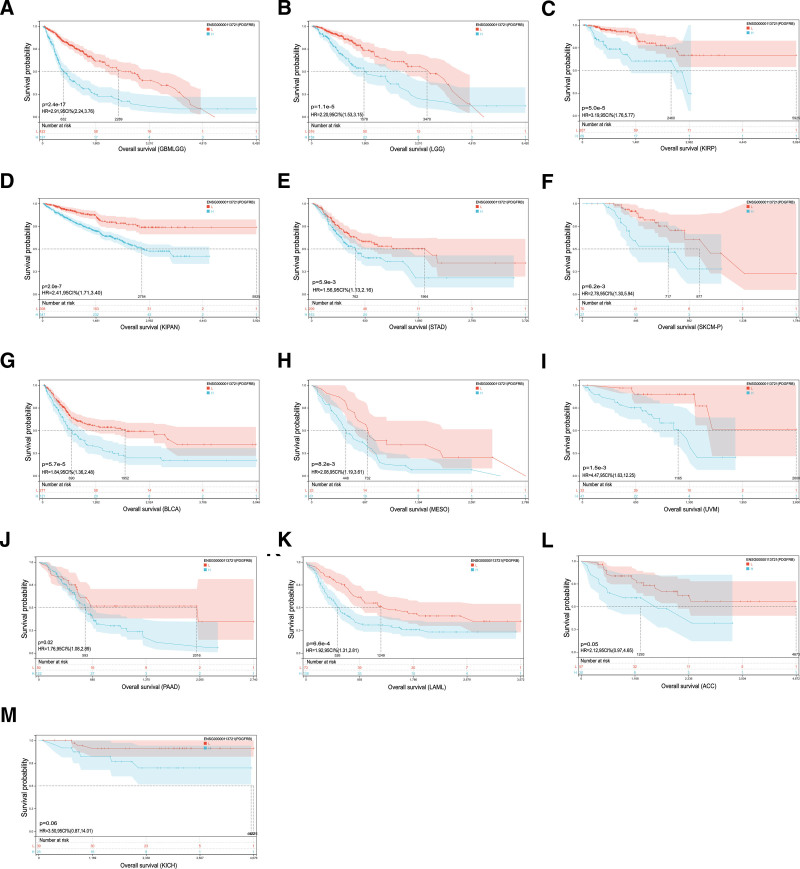
Kaplan–Meier overall survival rate for PDGFRB. The overall Kaplan–Meier survival rate of PDGFRB in tumor types is shown in the TCGA and GTEx databases. The median value of PDGFRB in each tumor was used as the critical value. (A–M). GTEx = Genotype-Tissue Expression, PDGFRB = platelet-derived growth factor receptor-β, TCGA = The Cancer Genome Atlas.

**Figure 5.**
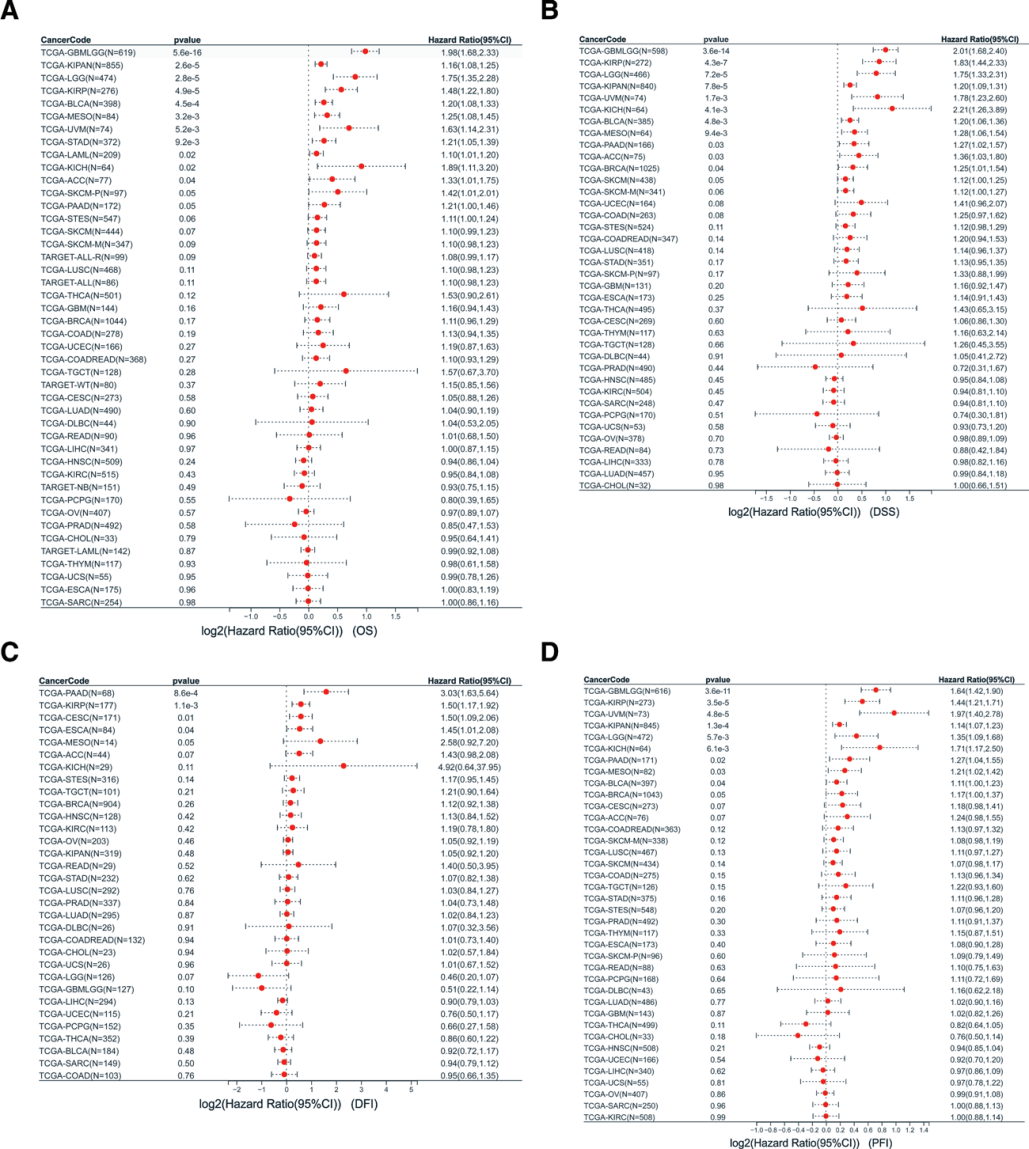
Univariate Cox regression analysis of PDGFRB. (A) The forest map shows the results of the OS on PDGFRB. (B) The forest map shows the results of the DSS on PDGFRB. (C) The forest map shows the results of the DFI on PDGFRB. (D) The forest map shows the results of the PFI on PDGFRB. DFI = disease-free interval, DSS = disease-specific survival, OS = overall survival, PDGFRB = platelet-derived growth factor receptor-β, PFI = progression-free interval.

The prognostic significance of PDGFRB in cancer patients was assessed by analyzing 13 different types of cancer, revealing significant differences in overall survival rates. Kaplan–Meier OS analysis results show that PDGFRB revealed significant prognostic differences in GBMLGG, LGG, KIRP, KIPAN, STAD, SKCM-P, BLCA, Mesothelioma (MESO), Uveal Melanoma (UVM), PAAD, and LAML but not in ACC and KICH (Figs. 4A–M). Univariate Cox regression analysis of OS indicated that PDGFRB was a risk factor for patients with GBMLGG, KIPAN, LGG, KIRP, BLCA, MESO, UVM, STAD, LAML, KICH, and ACC (Fig. 5A). DSS analysis revealed that PDGFRB is a risk factor for patients with several cancer types, including GBMLGG, KIRP, LGG, KIPAN, UVM, KICH, BLCA, MESO, PAAD, ACC, and BRCA (Fig. 5B). DFI analysis indicated that PDGFRB poses a risk for patients with PAAD, KIRP, and CESC (Fig. 5C). Furthermore, PFI analysis showed that PDGFRB is a risk factor for patients with GBMLGG, KIRP, UVM, KIPAN, LGG, KICH, PAAD, MESO, and BLCA (Fig. 5D).

#### 4.2. Analysis of tumor stemness and gene expression of PDGFRB

**Figure 6.**
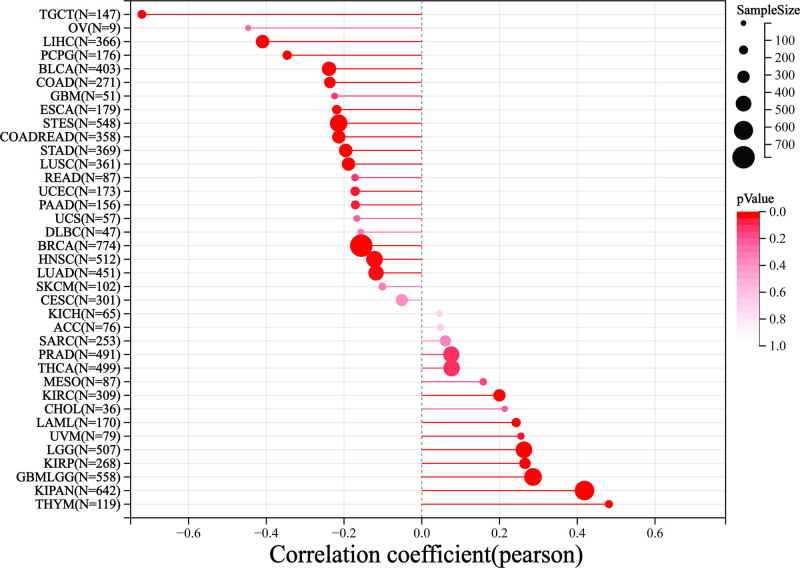
Analysis of tumor stemness and PDGFRB gene expression. PDGFRB = platelet-derived growth factor receptor-β.

The stemness index was integrated with the gene expression data from the samples. A log2 transformation (log2(x + 0.001)) was applied to each expression value, and cancer types with fewer than three samples were excluded, resulting in data for 37 cancer types. The analysis of the Pearson correlation coefficient showed significant positive correlations in 23 types of tumors. Among these, eight tumors exhibited particularly strong correlations: GBMLGG, LGG, LAML, KIRP, KIPAN, KIRC, THYM, and UVM. This indicates that a higher tumor stemness index is associated with greater expression of PDGFRB, which correlates with a worse prognosis. Conversely, the analysis found significant negative correlations in 15 tumors: LUAD, COAD, COADREAD, BRCA, ESCA, STES, STAD, UCEC, HNSC, LUSC, LIHC, PAAD, TGCT, Pheochromocytoma and paraganglioma (PCPG), and BLCA (Fig. 6).

### 5. The potential of PDGFRB as a biomarker for tumor immunotherapy

#### 5.1. Immune cell infiltration analysis of PDGFRB

**Figure 7.**
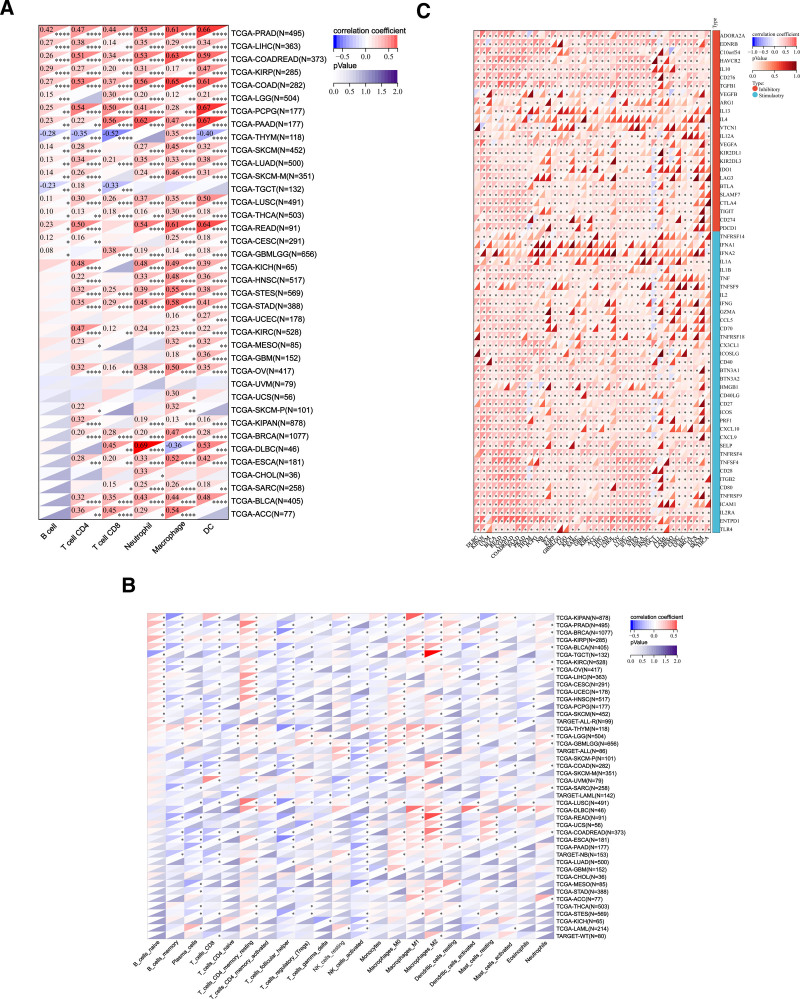
Immunocyte infiltration analysis of PDGFRB. (A) The TIMER database analyzed the correlation between PDGFRB and the infiltration level of B cells, T cells, neutrophils, etc. (B) Using data from the SCIBERSORT database, to investigate the correlation between PDGFRB and indicator immune cell infiltration levels. (C) PDGFRB immunologic checkpoint gene analysis. PDGFRB = platelet-derived growth factor receptor-β.

By analyzing the data from the TIMER database, scores for the infiltration of 6 types of immune cells were obtained from 9406 tumor samples from a total of 38 tumor types. PDGFRB immune cell infiltration scores were calculated for each tumor using the R software package. A total of 37 types of cancer were observed. PDGFRB expression in ACC, BLCA, BRCA, CESC, CHOL, COAD, COADREAD, Lymphoid Neoplasm Diffuse Large B-cell Lymphoma, ESCA, GBM, GBMLGG, HNSC, KICH, KIPAN, KIRC, KIRP, LGG, LIHC, LUAD, LUSC, MESO, OV, PAAD, PCPG, PRAD, READ, Sarcoma (SARC), SKCM-M, SKCM-P, SKCM, STAD, STES, TGCT, THCA, Thymoma (THYM), UCEC, and UCS was significantly correlated with immune infiltration (Fig. 7A).

Using the “CIBERSORT” method, immune cell infiltration scores were obtained for 22 types of immune cells from 10,180 tumor samples across 44 tumor types. The R software package was used to calculate the PDGFRB and immune cell infiltration scores. A total of 37 different types of cancer were observed. PDGFRB expression in GBM, GBMLGG, LGG, UCEC, LAML, BRCA, CESC, LUAD, ESCA, STES, SARC, KIRP, KIPAN, COAD, COADREAD, PRAD, STAD, HNSC, KIRC, LUSC, THYM, LIHC, High-Risk Wilms Tumor, SKCM-P, SKCM, BLCA, SKCM-M, THCA, Neuroblastoma, MESO, READ, OV, UVM, PAAD, TGCT, UCS, LAML, ALL, PCPG, ACC, ALL-R, Lymphoid Neoplasm Diffuse Large B-cell Lymphoma, and KICH significantly correlated with immune infiltration (Fig. 7B).

#### 5.2. Immune checkpoint analysis of PDGFRB

PDGFRB showed a positive correlation with immunosuppressive genes and immune activities, including IL13, IL4, IFNA1, and IFNA2. (Fig. 7C).

#### 5.3. T cell exhaustion analysis

**Figure 8.**
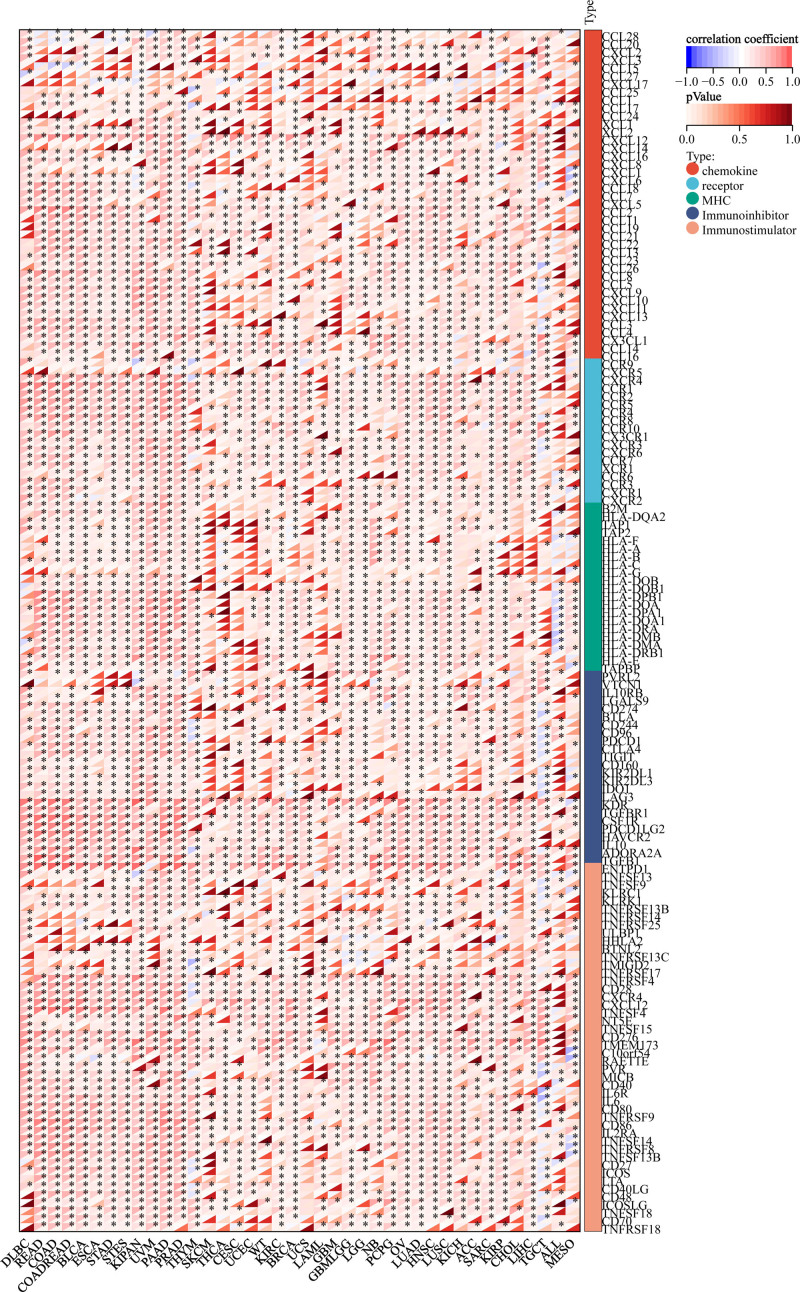
PDGFRB correlations between genes involved in immune regulation. The correlations between PDGFRB and chemokines, receptors, major histocompatibility antigens, immunosuppressive status-related genes, and immune activation status-related genes were shown from top to bottom. PDGFRB = platelet-derived growth factor receptor-β.

The study revealed significant relationships between PDGFRB and the major histocompatibility complex (MHC), as well as various genes that activate and suppress the immune system. It also examined the roles of chemokines and chemokine receptors. The results demonstrated that PDGFRB expression was positively correlated with several chemokines and their receptors, including CCL19, CCL21, CCR6, and CXCR6. Furthermore, PDGFRB showed a positive association with MHC, immune-activating genes, and immunosuppressive genes across different cancer types. Notable examples include TAP1, TAP2, CD28, CXCR4, and LAG3 (Fig. 8).

## 6. Discussion

This study presents a comprehensive pan-cancer analysis of PDGFRB, demonstrating that this gene exhibits significant differences in expression across various types of cancer. Moreover, PDGFRB is strongly associated with the prognosis of cancer patients. Notably, the research reveals a significant correlation between PDGFRB and immune response, particularly in the context of immune cell infiltration. In conclusion, we propose considering PDGFRB as a potential immune checkpoint. Pan-cancer research has recently gained significant attention due to its unique advantages. Previous studies primarily focused on individual cancers and single genes, which resulted in certain limitations. In contrast, pan-cancer research emphasizes the roles of single genes across various cancers, allowing for a more comprehensive understanding of how single genes contribute to different cancer types. This approach aims to yield more practical and valuable results.

The expression of ligands and receptors in the PDGF family varies according to the tumor tissue subtypes.^[20]^ In this study, we first assessed the expression levels of PDGFRB across various cancers. Our findings revealed that PDGFRB was significantly upregulated in 13 types of tumors, including GBM, LGG, and others. Conversely, it was significantly downregulated in 17 types of tumors, such as UCEC, BRCA, and CESC, among others. These results provide strong evidence of notable variations in PDGFRB expression across different cancer types. Additionally, previous research indicates that PDGFRB protein expression is significantly elevated in cases of congenital liver fibrosis.^[21]^ Then we looked at how PDGFRB gets expressed in different clinical stages. The results showed significant differences among the nine tumors, including COADREAD and ESCA.

In addition to its expression at different clinical stages, the differential expression of PDGFRB in tumor and standard tissue samples was analyzed. In 11 types of cancer, significant differences in the expression of PDGFRB were observed, particularly in UCEC, COAD, STAD, ESCA, and CHOL. A *t* test was conducted to analyze the expression in endometrial and colon cancers. The *P* value for endometrial cancer was .0000034, with a degree of freedom of 20, while the *P* value for colon cancer was .000029, with a degree of freedom of 40. Additionally, immunohistochemical staining was performed on tissue samples from ESCA and CHOL, as well as adjacent tissue samples. Although significant differences in expression were observed, trends could not be established due to a shortage of samples. Therefore, the preliminary significance of PDGFRB in tumor detection can be inferred.

The above research highlights the importance of PDGFRB in pan-cancer studies. This study then assessed the prognostic value of PDGFRB expression in cancer patients. The Kaplan–Meier curve is commonly used in clinical research, analysis results indicated that PDGFRB could be used to observe significant prognostic differences in GBMLGG, LGG, KIRP, KIPAN, STAD, SKCM-P, BLCA, MESO, UVM, PAAD, and LAML, but not in ACC and KICH. Our interpretation of the Kaplan–Meier analysis revealed that PDGFRB is a risk factor in cancer patients. OS results for Univariate Cox regression analysis revealed that PDGFRB was a risk factor for GBMLGG, KIPAN, LGG, KIRP, BLCA, MESO, UVM, STAD, LAML, KICH, and ACC. DSS analysis showed that PDGFRB was a risk factor for GBMLGG, KIRP, LGG, KIPAN, UVM, KICH, BLCA, MESO, PAAD, ACC, and BRCA.

Using OS as an endpoint in clinical studies may limit their feasibility, and deaths from causes unrelated to cancer can result in misleading outcomes. Additionally, both overall survival and DSS necessitate longer follow-up periods. Therefore, DFI or PFI may more accurately indicate the influence of various factors on patients in many clinical trials.^[22]^ To evaluate the correlation between PDGFRB and DFI or PFI in patients with tumors, this study also performed univariate Cox regression analysis. DFI analysis showed that PDGFRB was a risk factor for PAAD, KIRP, and CESC patients; PFI analysis showed that PDGFRB was a risk factor for GBMLGG, KIRP, UVM, KIPAN, LGG, KICH, PAAD, MESO, BLCA patients. In summary, PDGFRB is identified as a risk factor, with its high expression contributing to tumorigenesis. Previous studies have demonstrated a significant correlation between increased PDGFRB expression and the prognosis of breast cancer patients. In univariate analysis, high PDGFRB expression was associated with a shorter survival period. Additionally, in multivariate survival analysis, elevated PDGFRB expression serves as a crucial marker of poor prognosis.^[23]^ Some studies have also proposed a mechanism of PDGFRB in the poor prognosis of patients with gastric cancer. That is, high expression of PDGFRB can harm the prognosis of gastric cancer by promoting tumor angiogenesis and regulating the tumor immune microenvironment.^[14]^

Tumor stemness refers to the traits of tumor cells that resemble those of stem cells. Generally, a higher stemness in tumor cells correlates with increased invasiveness of the tumor and a poorer prognosis. This study analyzes PDGFRB in relation to tumor stemness, providing insights not only into prognosis but also evaluating the potential of PDGFRB as a marker for tumor stem cells. The results showed that PDGFRB was positively correlated with eight tumors, such as GBMLGG and LGG, and negatively associated with 15 tumors, such as PCPG and LUAD. GBMLGG is a type of multiform glioblastoma. In addition to the differential expression of PDGFRB in this tumor, previous studies have shown that another cancer gene, ERO1A, is significantly upregulated in GBMLGG.^[24]^ Glioma is the most common malignant primary brain tumor, known for its poor prognosis. Some studies suggest that an increase in tumor-associated macrophages (TAMs) may facilitate immune escape in glioma, making TAM-related signals valuable biomarkers for prognosis.^[25]^ Their research also offers insights for future studies on the role of PDGFRB in individual tumors. PCPG refers to pheochromocytoma, a neuroendocrine tumor that arises from the adrenal medulla or extra-adrenal ganglia.^[26]^

Cancer is closely linked to the immune system. This study analyzed the immune cell infiltration related to PDGFRB. Results from the TIMER database showed that PDGFRB expression was significantly correlated with immune infiltration in 37 types of cancer., including ACC, BLCA, and BRCA. CIBERSORT analysis showed that the PDGFRB expression was significantly correlated with immune infiltration in 43 cancer species, such as GBM, GBMLGG, and LGG. The above 2 methods of immune cell infiltration by PDGFRB have significantly demonstrated their correlation with immune infiltration. A promising approach in tumor immunotherapy is the blocking of immune checkpoints,^[27]^ Several immune checkpoint inhibitors (ICIs) have been developed to reactivate the anti-tumor immune response. These inhibitors work by blocking the co-inhibition signaling pathway and promoting the immune-mediated clearance of tumor cells.^[28]^ This study examined the immune checkpoint associated with PDGFRB. The results demonstrated a positive correlation between PDGFRB and immunosuppressive genes, including IL13 and IL4, as well as immune activation genes, such as IFNA1 and IFNA2.

Tumors select specific immune checkpoint pathways as immune resistance mechanisms, especially against tumor antigen-specific T-cells. Ligand-receptor interactions are the initiators of many immune checkpoints, which can be readily blocked by antibodies or modulated by recombinant forms of ligands or receptors.^[27]^ T-cell depletion is the loss of T-cell function in patients with chronic infections and cancer. A new mechanism of T cell exhaustion has been discovered, and a new strategy to restore T cell function by lowering cholesterol has been proposed to enhance T cell-based immunotherapy.^[29]^ Therefore, we carried out T cell exhaustion analysis of PDGFRB, and showed that PDGFRB expression correlated positively with chemokines and chemokine receptors. PDGFRB was also positively associated with MHC, immune-activating genes, and immunosuppressive genes in pan-cancers. These findings demonstrated the potential of PDGFRB as an immune checkpoint. Immune checkpoint inhibitors (ICIs) are considered a breakthrough in cancer treatment. However, only a small proportion of patients benefit from ICIs, which is a limitation of ICIs.^[30]^

Overall, this study analyzes PDGFRB across various cancer types, emphasizing its prognostic and immunological significance and potential role as an immune checkpoint. The findings provide a theoretical foundation for future research and suggest a new therapeutic direction for patients undergoing immunotherapy.

## Author contributions

**Conceptualization:** Feng Gao, Weizhong Huangfu, Minjie Wang.

**Data curation:** Qian Gao, Minjie Wang.

**Funding acquisition:** Minjie Wang.

**Investigation:** Yan Cui, Feng Gao.

**Methodology:** Qian Gao.

**Software:** Yan Cui.

**Supervision:** Yan Cui, Weizhong Huangfu.

**Validation:** Qian Gao, Yan Yang.

**Writing – original draft:** Qian Gao, Feng Gao.

**Writing – review & editing:** Yan Yang, Minjie Wang.
